# Investigating paternal preconception risk factors for adverse pregnancy outcomes in a population of internet users

**DOI:** 10.1186/s12978-016-0156-6

**Published:** 2016-04-14

**Authors:** Eleonora Agricola, Francesco Gesualdo, Emanuela Carloni, Angelo D’Ambrosio, Luisa Russo, Ilaria Campagna, Elisabetta Pandolfi, Alberto E. Tozzi

**Affiliations:** Multifactorial Disease and Complex Phenotype Research Area, Bambino Gesù Children Hospital IRCCS, Piazza S. Onofrio 4, 00165 Rome, Italy

**Keywords:** Preconception health, Preconception care, Adverse pregnancy outcomes, Maternal and child health, Preconception men, Paternal health, Men Health

## Abstract

**Background:**

Paternal preconception risk factors such as smoking, exposure to environmental substances, medication use, overweight and advanced age correlate with the occurrence of malformations and birth defects in the offspring. Nonetheless, the prevalence of risk factors for adverse pregnancy outcomes in the male population has been scarcely investigated and no report on preconception interventions targeting prospective fathers is available. We conducted a web-based survey to measure the prevalence of paternal preconception risk factors for adverse pregnancy outcomes in an Italian population of Internet users.

**Methods:**

Prospective or expectant fathers were enrolled during a four-week period through two of the main Italian web-sites dedicated to preconception, pregnancy, childhood and family care. Participants filled in a web questionnaire regarding preconception risk factors for adverse pregnancy outcomes. Logistic regression analysis was used to explore the predictors of paternal preconception risk factors.

**Results:**

We enrolled 131 prospective and 205 expectant fathers. More than half of the total participants used medications during the preconception period, 35 % were smokers and 8 % were obese. Exposure to environmental substances was declared by almost 20 % of the participants, with the group including pesticides/herbicides/professional paints being the most prevalent. More than a half of the study sample included men aged over 35 years.

According to the multivariate analysis, smoking and exposure to environmental toxics were less frequent among individuals with a university degree (respectively: OR = 0.52; 95 % CI 0.32–0.84; OR = 0.52; 95 % CI 0.29–0.93). Paternal obesity and medication use in the preconception period were not associated with any of the independent variables.

**Conclusions:**

The prevalence of preconception risk factors among male population should not be neglected when planning preconception interventions, confirming that preconception health must be focused on the couple, rather than on women only.

**Electronic supplementary material:**

The online version of this article (doi:10.1186/s12978-016-0156-6) contains supplementary material, which is available to authorized users.

## Background

Preconception interventions have been shown to improve the maternal health status and, in some cases, have been demonstrated to be effective in limiting the incidence of adverse pregnancy outcomes (APOs) [1, 2]. Until now, preconception health has mainly targeted women. Paternal preconception health has mainly been addressed as an opportunity to preserve the quality of germ cells and fertility, to improve men’s health before conception and to facilitate a better maternal health status during pregnancy [3–5]. Recently, paternal preconception health and lifestyles have been also correlated to the occurrence of malformations and birth defects in the offspring. Specifically, paternal preconception smoking, exposure to environmental substances, medication use, overweight and advanced age have been proved to be associated with low birth weight, congenital cardiac and anorectal malformations, infant cancers and neural tube defects (NTD) [6–12]. It has been proposed that prospective fathers should be targeted in preconception interventions as well as future mothers [3–5, 13, 14]. Nevertheless, to our knowledge, no actual intervention targeting prospective fathers has ever been reported in the medical literature.

Health professionals are encouraged to offer counselling to the prospective fathers at the time of preconception [15], nonetheless, the occasions of a direct involvement of men in such a context are still rare [5]. Although sociodemographic features and needs of men at high risk for APOs have been recently investigated in USA and Norway [16, 17], the prevalence of paternal preconception risk factors for APOs has been scarcely studied [4]. As a matter of fact, these figures could be inferred from studies on risk factors for other diseases conducted in the general male population. However, such studies are not specifically targeted on men in the preconception period.

To our knowledge, in Italy, no study was ever conducted to investigate this issue.

We conducted a web-based survey to measure the prevalence of paternal preconception risk factors for APOs in a population of Italian Internet users.

## Methods

### Study design, study population and definitions

We conducted a cross sectional, web-based survey to measure the prevalence of paternal preconception risk factors for APOs in a population of Italian Internet users, who were either prospective or expectant fathers. We defined a) a prospective father as a man of a couple planning a pregnancy in the following year; b) an expectant father as a man of a couple with an ongoing pregnancy.

The study was conducted during June 2014, for four weeks.

We identified a set of paternal preconception risk factors for APOs after reviewing the literature. On this basis, we set up an anonymous questionnaire through the Survey monkey web platform (it.surveymonkey.com). The questionnaire included the following items: a) social and demographic data: age (continuous), education level (university degree or lower), employment (employed-not employed); b) risk factors for APOs: being obese (BMI ≥ 30); smoking (any quantity); use of medications in the previous three months or in the three months before conception (prescribed and over the counter medications); occupational or hobby exposure to environmental substances (pesticides, herbicides, professional paints, dry cleaning products; lead; exhaust fumes; textiles, rubber, wood, paper and printing industry products), advanced age (> = 35 years old) (Additional file 1).

We selected two of the main Italian web-sites dedicated to preconception, pregnancy, childhood and family care (www.nostrofiglio.it; www.periodofertile.it). These websites are monthly visited by 1–3 million unique visitors, mainly between 25 and 44 years of age, living across the country. The social networks of the websites are also intensely visited.

Within the selected websites, we set up a web page presenting the study and including a link to the web questionnaire. After accessing the questionnaire web page, we screened potential participants for inclusion criteria: male gender of respondents, being 18 years of age, residence in Italy, partner with an ongoing pregnancy or pregnancy planned in the following year. In case the couple was already expecting a baby, questions were anyway referred to paternal risk factors before conception.

If eligibility criteria were not met, the survey was not accessible. Informed consent to participation was then obtained through an online form. Participants were never interviewed in person and data were collected anonymously. The system did not allow to fill in the questionnaire more than once.

We promoted the project through the webpages, the social network accounts (Facebook and Twitter) and the newsletters of the websites hosting the study. No paid advertising was used and no incentives were offered for participation.

The study was approved by the Bambino Gesù Children’s Hospital’s Ethical Committee.

### Statistical analysis

We described socio-demographic variables and risk factors as means and standard deviations (SD) or proportions and 95 % confidence intervals (CI), as appropriate. We compared the different proportions between expectant fathers and prospective fathers using Chi-square test or Fisher exact test, while differences of means were studied through Student’s *T*-test. After verifying absence of multicollinearity by studying the correlation matrix and the variance inflation factor, through multivariate logistic regression we explored how the prevalence of risk factors, adjusted for age, was affected by the following independent variables: pregnancy status (planned or ongoing pregnancy), education level, employment. The risk factors studied as dependent variables in the multivariate analysis were: being obese, exposure to environmental substances, use of medications, smoking. We did not include advanced age in the model as it is not a modifiable risk factor. Results were considered statistically significant if the *p*-value was <0.05. We calculated the sample size on the basis of the prevalence of smokers. In order to measure a prevalence of 30 % with a precision of ±5 % and a confidence level of 95 %, a population of 323 individuals was required. We used the STATA 12 statistical package to perform the statistical analysis.

## Results

A total of 336 men filled in the questionnaire, of whom 131 (39 %) were prospective fathers planning a pregnancy and 205 (61 %) were expectant fathers.

Table 1 shows the socio-demographic characteristic of the enrolled population. The majority of the participants reported to be employed and more than 41 % reported to be graduated.

**Table 1:** Characteristics of men included in the study

	Total	Prospective Fathers	Expectant Fathers	*p*
	*N* = 336	*N* = 131	*N* = 205	
Mean age, yrs (SD)	35.27 (7.18)	35.06 (7.33)	35.40 (7.10)	0.677
University Degree, *N* (%)	140 (41.67)	53 (40.46)	87 (42.44)	0.719
Employed, *N* (%)	315 (95.17)	125 (96.90)	190 (94.06)	0.240

The prevalence of the paternal preconception risk factors for APOs is presented in Table 2. More than a half of the study sample included men aged over 35 years. Almost eight percent of participants were obese and more than one third were smokers. Almost 20 % reported to be exposed to teratogenic environmental substances, with the group including pesticides/herbicides/professional paints being the most prevalent. More than a half of the participants used medications during the preconception period. Most used drugs were anti-inflammatory and anti-rheumatic products and analgesics products (data not shown).

**Table 2:** Prevalence of paternal preconception risk factors, by paternal status regarding pregnancy

	Total	95 % CI	Prospective Fathers	Expectant Fathers	*p*
	*N* = 336 (%)		*N* = 131 (%)	*N* = 205 (%)	
Age ≥ 35	176 (52.54)	47.16–57.91	65 (49.62)	111 (54.41)	0.391
BMI ≥ 30	26 (7.74)	4.87–10.61	9 (6.87)	17 (8.29)	0.634
Medication use	174 (51.94)	46.56–57.32	76 (58.02)	98 (48.04)	0.075
Smoking, any quantity	118 (35.44)	30.27–40.60	40 (30.53)	78 (38.61)	0.132
Exposure to environmental substances	65 (19.35)	15.10–23.59	24 (18.32)	41 (20.00)	0.704
pesticides/herbicides/professional paints	28 (8.33)	5.36–11.30	11 (8.40)	17 (8.29)	0.973
lead/exhaust fumes	24 (7.14)	4.38–9.91	9 (6.87)	15 (7.32)	0.877
textiles, rubber, wood, paper and printing industry products	26 (7.74)	4.87–10.61	12 (9.16)	14 (6.83)	0.435
professional dry cleaning products	1 (0.30)	−0.29–0.88	0 (0.00)	1 (0.49)	0.423

As shown in Table 3, the multivariate analysis showed the following: smoking was less frequent among individuals with a university degree (OR = 0.52; 95 % CI 0.32 – 0.84); exposure to environmental toxics was less frequent among individuals with a university degree (OR = 0.52; 95 % CI 0.29– 0.93). Paternal preconception obesity and medication use in the preconception period were not associated with any of the independent variables.

**Table 3:** Determinants of paternal preconception risk factors for adverse pregnancy outcomes

Risk factors	Pregnancy		Education level		Employment	
	OR (95 % CI)	*P*	OR (95 % CI)	*P*	OR (95 % CI)	*P*
BMI ≥ 30	1.28 (0.53–3.10)	0.582	0.70 (0.29–1.69)	0.422	0.57 (0.12–2.79)	0.489
Medication use	0.64 (0.41–1.01)	0.055	0.87 (0.56–1.36)	0.538	0.93 (0.33–2.63)	0.898
Smoking, any quantity	1.45 (0.90–2.35)	0.130	0.52 (0.32–0.84)	0.008	0.60 (0.20–1.75)	0.347
Exposure toenvironmental substances	1.12 (0.63–1.98)	0.692	0.52 (0.29–0.93)	0.029	1.48 (0.39–5.61)	0.566

## Discussion

This study shows a high prevalence of paternal preconception risk factors for APOs in a population of Italian Internet users. These results provide information on the characteristics of men who most need further attention by health professional in the preconception period.

The evidence for the association between paternal preconception smoking and acute lymphoblastic leukemia [6, 18], anorectal malformations [9] and congenital cardiac malformations [19] is robust. A recent study suggests that paternal smoking in childhood may lead to obesity in the offspring during adolescence [20]. We found a considerably high prevalence of smokers in our study population (35 %). Such figure is higher compared both to the prevalence of smokers in the general male population of the same age group (26 %) (http://dati.istat.it) and to figures regarding a group of Italian women of childbearing age planning a pregnancy (16 %) [21]. This figure is worrisome, as it suggests that, during pregnancy, women are likely to be exposed to passive smoking, which represents a risk factor for low birth weight [22]. Several studies showed that women are likely to quit smoking before pregnancy [23, 24] and that women’s success to quit depends on the partner’s smoking cessation [25, 26]. Therefore, preconception counselling and information campaigns promoting smoking cessation should also target men, addressing in particular those with a lower level of education. In our population, in fact, men with a lower level of education had a higher risk of smoking, which confirms previous evidence [27].

The evidence of the association between exposure to environmental substances and APOs has been repeatedly demonstrated [28]. Lead, organic solvents and pollutants, exhaust fumes and pesticides are associated with low birth weight and with an increased risk of malformations [7–9, 29–32]. Childhood cancers are associated with fathers’ exposure to professional paints, pesticides and products containing pentachlorophenol [28, 33–37]. Furthermore, an increased risk of neural tube defects has been demonstrated for specific job categories [38]. Notably, in our population, the exposure to pesticides and herbicides is quite high.

Regarding medications, there is a scarce knowledge of the effects of paternal preconception medication use on the offspring. Mercaptopurine and azatiopurine, anti-inflammatories, antihypertensives, antipsychotics and drugs for acid-related disorders, when used in the preconception period, are associated with the onset of pregnancy complications and birth defects, including hypospadias [32, 39]. Contradictory results have emerged from other studies [39]. Recently, preconception use of diazepam by prospective fathers was shown to be associated with an increased risk of perinatal mortality and growth retardation, nonetheless no association was found between the intake of other drugs 3 months before conception and occurrence of APOs [10]. In some European countries, an extensive use of medicines by men in the preconception period has been documented [39], and the services for teratology information often offer counselling regarding drug assumption to expectant fathers [40, 41]. In particular, in Italy, the questions most frequently received by the Italian Teratology Information Service regarding paternal exposure are those concerning drug exposure [40], being diagnostic radiations, recreational drugs and occupational chemicals other items of interest [40]. In our study, we found that the prevalence of medication use is high, similarly to figures reported by other countries [10, 39]. The results of the present study consolidate the view that more studies are needed to understand the relationship between APOs and drug intake in men in the preconception period.

Epidemiological evidence suggests that obesity of perspective fathers may induce epigenetic changes in fetal genes involved in the regulation of the early stages of the embryonic growth [11, 42, 43]. Paternal obesity has been also associated with autism spectrum disorders [44]. In our study population, the proportion of overweight men is slightly lower than that of the general male population (7.7 vs 11.5 %) (http://dati.istat.it).

Although the definition of paternal advanced age diverges across the literature, several authors have associated it with adverse fetal outcomes, including birth defects, low birth weight, autism, schizophrenia [12, 45]. In our study, more than a half of the participants are men aged over 35, consistently with the general male population, whose mean age at the time of the first child is 35.80, as reported by the Italian Institute of Statistics (http://dati.istat.it).

Our results suggest that preconception counselling and information campaigns should be focused both on women and men. Health professionals should carefully communicate the risk factors associated with postponed pregnancy and strongly recommended the couple to modify at-risk behaviors, such as smoking. An occupational medicine specialist should be involved during the preconception counselling and specific behaviors to reduce the accidental transfer of chemicals to domestic spaces should be recommended. However, every health professional should receive a proper education on this issue [46], which is often neglected both by the public and by health professionals. Information campaigns regarding the proper use of medications and the effect of drug intake on the offspring should be implemented; to this regard, pharmacists should also be actively involved [47]. Taking into account the risks of preconception paternal obesity on the offspring, a healthy diet and a regular physical exercise should also be actively promoted.

In the last years, men’s responsibility for their own reproductive health has been emphasized [48]. As reaching and involving men in reproductive health is a challenging task, World Health Organization (WHO) has issued specific recommendations on this issue (http://apps.who.int/iris/bitstream/10665/67409/1/WHO_FCH_RHR_02.3.pdf).

Firstly, national policies should include men in reproductive health services and programs focusing on specific men’s health needs. Secondarily, interventions, services and campaigns should specifically target the male population, addressing men separately from women. To effectively achieve this objective, such strategies should be implemented through the existing channels within the men’s communities (http://apps.who.int/iris/bitstream/10665/67409/1/WHO_FCH_RHR_02.3.pdf).

Although traditional health promotion strategies remain highly efficacious [49], evidence exists that eHealth interventions positively impact on health-related lifestyle and knowledge [50], and therefore could effectively integrate traditional health promotion strategies.

eHealth interventions dedicated to sexual and reproductive health [51–53], including preconception healthcare [21, 23], have been previously described and should be further investigated, in order to better characterize their potential in reaching and informing the men, including those rarely attend medical visits.

The present study has a number of limitations. Information provided by expectant fathers could suffer from recall bias and information bias: if a pregnancy is ongoing, a riskful preconception lifestyle could be hidden for fear of social stigma.

The link to the questionnaire was published on websites mainly visited by women. When designing the study, we explored the possibility of linking the survey on websites dedicated to men. Nevertheless, we realized that the impact of such a promotion strategy could have been very limited, as men’s health was poorly addressed on popular Italian men websites. Moreover, preconception health is, in general, a neglected issue among the Italian population (R. Bortolus personal communications) and on the web [54]. In order to improve the response rate, we decided to promote the survey on websites where the users were likely aware of and interested in this subject. However, this choice might have introduced a selection bias, causing an underestimation of the risk factor prevalence compared to the general population.

Another selection bias might be due to the fact that enrolled individuals were either planning a pregnancy or had an ongoing pregnancy, therefore we excluded those couples that could be at risk of an unplanned pregnancy.

Another limitation of the study also regards the evaluation of teratogenic drugs among the study population, as the active principles of the used drug have not been explicitly declared by responders. Therefore, a precise characterization of the population taking teratogenic drugs has not been possible. Furthermore, we had no information regarding the pregnancy outcomes and the female partners’ health status and lifestyles, which are a crucial information for a proper analysis of preconception risk factors and outcomes in the offspring.

## Conclusions

Our study shows that the prevalence of preconception risk factors among male population should not be neglected when planning preconception interventions. Reducing paternal preconception risk factors might limit the burden of APOs. This confirms that preconception health must be focused on the couple, rather than on women only.

## Additional file

Additional file 1: Survey items on paternal preconception risk factor for APOs. (DOCX 16 kb)

## Abbreviations

ACOG: American College of Obstetricians and Gynecologists
APOs: adverse pregnancy outcomes
BMI: body mass index
WHO: World Health Organization
