# Supplementary material for: Investigating paternal preconception risk factors for adverse pregnancy outcomes in a population of internet users
Source: Reprod Health. 2016 Apr 14;13:37. doi: 10.1186/s12978-016-0156-6 (PMC4832499; doi:10.1186/s12978-016-0156-6)
Supplement: Additional file 1: — Survey items on paternal preconception risk factor for APOs. (DOCX 16 kb) [file 12978_2016_156_MOESM1_ESM.docx]

**Additional File 1: Survey items on paternal preconception risk factor for APOs**

1. Are you expecting a baby? *If NO, automatically directed to #2; if YES, automatically directed to #3*
2. Are you planning a pregnancy in the next 1 year?
3. How old are you?
4. Education
5. Employment
6. How much do you weight? (kg) *Only IF answered NO to #1 and YES to #2*

OR

How much did you weight, before conception? (kg) *Only IF answered YES to #1*

1. How tall are you? (cm)
2. Do you smoke? *Only IF answered NO to #1 and YES to #2*

OR

Did you smoke before conception? *Only IF answered YES to #1*

1. For hobby or professional reasons, are you exposed to: *Only IF answered NO to #1 and YES to #2*

OR

Before conception, for hobby or professional reasons, were you exposed to: *Only IF answered YES to #1*

- pesticides/herbicides/professional paints?
- lead/exhaust fumes?
- textiles, rubber, wood, paper and printing industry products?
- professional dry cleaning products?

1. In the last three months, have you taken any medicine (including over the counter medicine)? *Only IF answered NO to #1 and YES to #2*

OR

In the three months before conception, have you taken any medicine (including over the counter medicine)? *Only IF answered YES to #1*
